# Hearing aid fitting: analysis of the impact on the auditory system during the first year of use in moderate and severe hearing loss

**DOI:** 10.1590/2317-1782/e20250077en

**Published:** 2026-06-15

**Authors:** Victor Goiris Calderaro, Maria Stella Arantes do Amaral, Eduardo Tanaka Massuda, Ana Cláudia Mirândola Barbosa Reis

**Affiliations:** 1 Centro Especializado de Otorrinolaringologia e Fonoaudiologia, Hospital das Clínicas de Ribeirão Preto, Faculdade de Medicina de Ribeirão Preto – FMRP, Universidade de São Paulo – USP - Ribeirão Preto (SP), Brasil.; 2 Departamento de Oftalmologia, Otorrinolaringologia e Cirurgia de Cabeça e Pescoço, Faculdade de Medicina de Ribeirão Preto – FMRP, Universidade de São Paulo – USP - Ribeirão Preto (SP), Brasil.; 3 Departamento de Ciências da Saúde, Faculdade de Medicina de Ribeirão Preto – FMRP, Universidade de São Paulo – USP - Ribeirão Preto (SP), Brasil.

**Keywords:** Hearing Loss, Hearing Aids, Event-Related Potentials, P300, Speech Perception, Surveys and Questionnaires

## Abstract

**Purpose:**

To investigate the behavior of the auditory system in people’s first year of hearing aid use.

**Methods:**

The study included 24 individuals of both sexes with moderate and severe sensorineural hearing loss. The first data collection stage occurred before fitting the hearing aids, through the P300 electrophysiological examination and the Portuguese Sentence List test. The second and third stages occurred 6 and 12 months after starting rehabilitation, applying these two tests and the Hearing Handicap Inventory for Adults or Hearing Handicap Inventory for the Elderly.

**Results:**

The P300 latency and amplitude did not differ between the stages with either tone-burst or speech stimulus. The post-fitting hearing thresholds allowed accessibility to speech sounds, therefore differing between stage 1 and stages 2 and 3, with no difference between the last two. There was evidence of improved speech perception throughout the three phases, particularly in individuals with moderate hearing loss. The self-assessment questionnaire showed that the use of hearing aids reduced perceived participation restrictions in emotional and social contexts.

**Conclusion:**

Despite the lack of changes in electrophysiological measurements during the first year of use, speech perception (recognition) improved. Users also reported an improvement in quality of life, highlighting the benefits of using hearing aids.

## INTRODUCTION

Hearing loss impacts a person's life in their ability to perceive stimuli, relate to others, and convey ideas and feelings. Furthermore, studies have shown that the duration of hearing loss may be associated with cognitive decline and intermodal cortical reorganization, especially in older adults^(1,2)^. Following a diagnosis of hearing loss, the use of electronic hearing devices, including hearing aids (HAs), cochlear implants, and bone-anchored HAs, has proven to be an effective and promising intervention. HAs are the most widely used option worldwide, and their reintroduction for auditory stimulation can lead to functional changes in the system responsible for auditory information^(3)^. Objective tests have become popular for evaluating the auditory system and monitoring the rehabilitation process. Although no single procedure can answer questions about the functional integrity of the central auditory system^(4)^, the combination of objective and behavioral tests can provide information about the neural processes involved in decoding.

It is important to include electrophysiological hearing tests in clinical practice because auditory evoked potentials allow the evaluation of the entire auditory system, from the auditory sensory organ to the subcortical and cortical areas^(5)^.

The P300 long-latency auditory evoked potential (LLAEP) has been considered a promising tool for monitoring interventions, as it can be recorded in individuals with hearing loss who can access and discriminate stimuli^(6)^. The P300 is an electrophysiological marker related to cognitive processes, such as attention and auditory discrimination, and is analyzed by latency and amplitude measures^(5)^. In HA users, its analysis allows the investigation of the behavior of the central auditory system resulting from sound stimulation, complementing the traditional perceptual assessment. HA use can promote the plasticity of central auditory pathways 6 months after fitting, increasing the number of neurons responsive to sound stimuli^(7)^.

Along with electrophysiological tests, behavioral assessment of sentence speech perception allows us to verify auditory performance in communication situations. Quiet environments with a low signal-to-noise ratio favor the speech intelligibility of HA users. Thus, speech recognition tests with competing noise have been developed to assess people with hearing loss, as they verify auditory skills in conditions that resemble everyday life situations^(8)^.

Besides electrophysiological and behavioral tests, the combined use of questionnaires that assess how HA users perceive the restriction of participation in everyday situations can provide a detailed understanding of the effects of hearing loss on communication and quality of life^(9)^. Thus, using standardized questionnaires allows us to establish the possible needs and expectations of patients, quantify the improvement after the intervention^(10)^, and validate decisions in audiological practice.

Few studies in the literature have used the LLAEP P300 in patients with newly fitted bilateral HA. On the other hand, there is a trend towards combining behavioral and objective tests for monitoring. Considering the possibility of understanding the difficulties faced in fitting, reassessing them when necessary, and replanning the process, this study aimed to investigate how the auditory system behaves during the first year of bilateral HA use in adults with moderate to severe postlingual hearing loss, considering aspects of behavioral, electrophysiological, and self-reported auditory perception.

## METHODS

This is an observational, descriptive, longitudinal, comparative study with an emphasis on diagnostic research. It was approved by the research ethics committee of the Clinics Hospital of the Medical School of Ribeirão Preto, University of São Paulo, Brazil, under protocol number HCRP 8774/2017. Data collection procedures began only after all participants signed an informed consent form.

### Participants

The study included 24 individuals with bilateral, symmetrical, sensorineural hearing loss (18 moderate and six severe)^(11)^, of both sexes (11 men and 13 women), aged 30 to 78 years, with a mean age of 58.9 years (SD ±15.0). This age range was chosen to represent the diversity of HA users treated in a clinical setting. The age of hearing loss acquisition ranged from 9 to 65 years (mean 46.54 years), and the length of hearing deprivation ranged from 2 to 31 years (mean 12.37 years). Regarding the etiology of hearing loss, 21 of them were under investigation, one was diagnosed with noise-induced hearing loss, and two with presbycusis.

Inclusion criteria considered individuals aged 18 to 79 years, of both sexes, with moderate to severe bilateral, symmetrical, sensorineural hearing loss, with no history of using electronic hearing devices of any type. The exclusion criteria were individuals diagnosed with neurological and/or psychiatric disorders, conductive and/or mixed hearing loss, and those who did not wish to complete the assessment process. The 10-Point Cognitive Screening (10-CS) was used in individuals over 60 years old to control for exclusion criteria^(12)^. Although no specific tests of linguistic competence were applied, this cognitive screening included items of orientation and verbal comprehension, ensuring that participants had adequate cognitive and communicative conditions to perform the tasks.

### Stages and procedures

Participants were initially interviewed to obtain identification and demographic data, including age, education level, sociocultural level, and occupation, and answered the 10-CS. Clinical records were also consulted to obtain audiological information, including the classification of hearing loss, hearing deprivation time, and etiology of the loss. Then, research data were collected in three stages.

The first stage took place on the day the participants received bilateral HAs, prior to their fitting, and consisted of researching free-field hearing thresholds without HAs, applying free-field LLAEP P300, and applying the Portuguese Sentence Lists (LSP) test^(13)^. The second and third stages took place 6 and 12 months (± 1 month) after HA fitting, which were fitted with the NAL-NL2 prescriptive rule. The necessary measures were taken to ensure that participants received adequate amplification. All used it for at least 8 hours a day, according to self-report. This study did not use the HA record (datalog); therefore, it is suggested that future studies use the time record (datalog) in the programming software to record usage time accurately. These stages included the research of free-field hearing thresholds with and without HAs and the application of the free-field LLAEP P300 with HAs, the LSP test, and the Hearing Handicap Inventory for Adults (HHIA)^(14)^ or Hearing Handicap Inventory for the Elderly (HHIE)^(15)^, according to the person's age.

Free-field hearing thresholds were measured with the Madsen Astera 2-Otoflex audiometer and a loudspeaker inside the audiometric booth at 500, 1000, 2000, 3000, and 4000 Hz. During the procedure, the participant sat 80 cm away from the speaker, positioned at 0° azimuth, at ear level. Thresholds were investigated using the descending-ascending technique with a warble stimulus; they were asked to raise their hand upon hearing the stimulus.

The electrophysiological examination used the LLAEP P300, performed on the Navigator^®^ Pro equipment from Bio-logic^®^ Systems Corp., two-channel auditory evoked potential – AEPSystem, version 1.3.0/Natus Medical, USA, connected to a conventional computer. It used the following recording parameters: gain of 50,000 µV, filter 30.0 (high frequency) and 1.0 (low frequency), analysis time of 520 ms, and rarefied polarity. The electrode setup consisted of the active electrode positioned at Cz (jumper, channels 1 and 2, input 1), reference electrodes on the left (A1) and right (A2) earlobes (input 2), and a ground electrode at Fpz. An individual electrode impedance of ≤ 5 kΩ and an inter-electrode impedance of ≤ 2 kΩ were accepted.

The test was performed in a free field, in an acoustically controlled environment, with the participant semi-seated in a reclining chair. The speaker was placed 1 meter away, at 0º azimuth, at the height of the auricle.

Participants were instructed to keep their eyes open and fixed on a specific point in front of them to avoid artifacts, and to raise their index finger when listening to the stimuli, identifying only the rare stimulus. The test used tone-burst and speech stimuli at 30 dB HL to ensure the principles of acoustic stimulus perception (i.e., to enable the principle of equality to the condition of the participants regarding stimulus intensity). Thus, tone-burst stimuli were presented at 1000 Hz (frequent stimuli) and 2000 Hz (rare stimuli), 30 dB HL above the free-field psychoacoustic threshold for these frequencies. Speech stimuli were the syllables /ba/ (frequent stimuli) and /da/ (rare stimuli), already synthetically present in the equipment, presented at 30 dB HL, based on the three-tone average (500, 1000, and 2000 Hz) of the free-field psychoacoustic threshold.

Both stimuli were presented in random and unpredictable order, with a 20% probability of rare stimuli (40 stimuli) and an 80% probability of frequent stimuli (approximately 200 stimuli). Two successive screenings were applied to ensure reliable electrophysiological recordings and to replicate the waves. The relationship between identifying rare stimuli by raising the index finger and the actual appearance of the stimuli was also observed to rule out the lack of P300 recording due to the participant's inattention. The analysis used the recording with the best wave morphology.

The LSP test was used for speech perception analysis, comprising seven lists to measure speech recognition skills. One of the lists (1A) has 25 sentences, and the other six (1B, 2B, 3B, 4B, 5B, 6B) have 10 sentences each. The lists are similar in terms of phonetic context and sentence structure, making them equivalent. Words with lexical and semantic meaning, such as nouns, adjectives, and verbs, were considered keywords and scored with 2 points. Words with grammatical meaning, such as prepositions and conjunctions, score 1 point. Since the lists have different word counts, the total score was multiplied by a correction value, obtaining a maximum score of 100%^(13,16)^.

First, the sentence recognition threshold in quiet (SRTQ) was obtained, indicating the intensity required for the individual to recognize about 50% of the stimuli. The test was conducted in a controlled free-field environment, where the participant was seated in front of the sound source, at ear level, and at 0° azimuth. Sentences from list 1A were used at this point. The sentences were presented according to the ascending-sequential strategy to obtain the SRTQ^(17)^. Thus, once the individual responded correctly to an entire sentence, the following sentence was presented at a lower intensity. If he did not respond correctly, the intensity was increased. Initially, 10 dB intervals were used until the individual made a mistake, then 5 dB intervals were used until finding the SRTQ.

After finding each participant’s SRTQ, it was kept fixed to obtain the sentence recognition percentage index in quiet (SRPQ), which is the sum of all correctly repeated words in each of the 10 sentences, multiplied by the correction value to obtain a percentage number. The test used lists 1B (stage 1), 3B (stage 2), and 5B (stage 3), and scores ranged from 0% (minimum performance) to 100% (maximum performance).

In stage 3, in addition to SRTQ and SRPQ, the sentence recognition percentage index in noise (SRPN) was also calculated, using list 6B. In this test, noise was presented simultaneously with the sentences. The signal-to-noise ratio was kept at -5 dB, with the speech being presented at 0° and the noise at 180°. The intensity of the speech was the same as the SRTQ.

The HHIA/HHIE was applied during stages 2 and 3 to characterize participation restriction, since this questionnaire is only used when the individual is using electronic hearing devices. Its 25 questions are divided into two scales, one social/situational and the other emotional. The questionnaire was used as a guided script and administered as a direct interview; the participant was instructed to choose one answer for each question: yes (4 points), sometimes (2 points), or no (0 points). The total score was calculated by adding the points; the higher the score, the greater the self-perceived restriction. Less than 16 points meant no perceived participation restriction, 18 to 42 points meant a mild restriction, and above 42 points meant a severe or significant participation restriction.

### Data analysis

Qualitative results were presented as frequency and proportion. Quantitative results were presented as mean and standard deviation (mean ± SD) when parametric methods were used, and median (interquartile range [IQR]) when evaluated with non-parametric methods. We used repeated measures analysis of variance (ANOVA-RM) to compare differences in latency (ms) and amplitude (µV) between phases. Dependent measures were compared using the Wilcoxon test and Student's t-test. The Mann-Whitney and Kruskal-Wallis tests were used for independent measures. Correlations between variables were assessed using the Tukey-HSD test, or had the p-value corrected using the false discovery rate (FDR). The significance level was set at p < 0.05.

## RESULTS

The free-field hearing threshold research found average hearing thresholds for the three stages, being: Stage 1 = 55.1 dB SPL, Stage 2 = 36.1 dB SPL, and Stage 3 = 36.9 dB SPL. As expected, due to the benefit of using HAs, there is a statistical difference (p < 0.001) between stages 1 and 2 and stages 1 and 3 when using HAs. The comparison between stages 2 and 3 found similar results, with no statistical difference.

Table 1 shows the means and standard deviations of the P300 latency and amplitude measures for all 24 participants, in all stages, and for both stimuli. The latencies for speech stimuli were lower in all three stages than for the tone-burst stimuli; the amplitudes, however, were similar. Regardless of the type of stimuli, the latency averages are within the expected range as a normal standard for the P300.

**Table 1:** Mean latency (ms) and amplitude (µV) measures of the P300 potential in the three stages of the study for both tone-burst and speech stimuli

**Stimulus**	**Stages**	**Latency (ms)**		**Amplitude (µV)**	
		**Mean**	**SD**	**Mean**	**SD**
Tone-burst	1	348.85	51.5	3.38	2.6
	2	334.84	37.1	4.12	2.8
	3	337.01	40.6	4.35	2.2
Speech	1	326.08	27.6	4.25	2
	2	330.72	27.1	4.26	1.8
	3	318.62	29.6	3.70	2.1

**Caption:** SD = standard deviation; ms = milliseconds; µV = microvolts. Source: Developed by the authors

**Figure 1:**
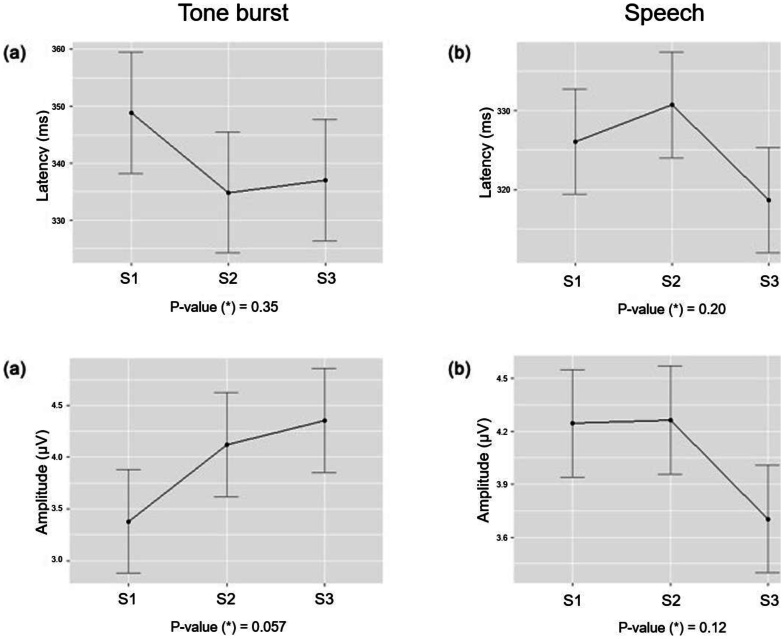
Mean P300 latency (ms) and amplitude (µV) measurement values for tone-burst (a) and speech (b) stimuli in the three stages of the study

Figure 1 shows the average P300 latency measures for all three stages, considering tone-burst and speech stimuli. Statistical analysis compared the stage without HAs with the stages in which they used HAs (S1 x S2 and S1 x S3) and indicated no statistical difference (p = 0.35 and p = 0.20, respectively). Similarly, the mean values ​​of the P300 amplitude measures using tone-burst and speech stimuli showed no difference (p = 0.057 and p = 0.12, respectively), as observed in Figure 1.

**Caption:** S1 = Stage 1, before hearing aid fitting; S2 = Stage 2, six months after hearing aid fitting; S3 = Stage 3, twelve months after hearing aid fitting; ms = millisecond; µV = microvolt. (*) = analysis of variance for repeated measures (ANOVA-RM). Source: Developed by the authors

An analysis was performed between the education level and the P300 latency and amplitude results, which showed no statistical difference (p-values ​​for speech stimuli, for latency and amplitude, respectively, in the three stages were p = 0.49; p = 0.86; p = 0.8 and p = 0.49; p = 0.86; p = 0.81. For tone-burst stimuli, they were p = 0.91; p = 0.04; p = 0.06 and p = 0.79; p = 0.10; p = 0.54). Furthermore, there was no statistical evidence of a correlation between P300 latency or amplitude and the length of auditory deprivation in the participants for the three stages (p-values ​​for speech stimuli, for latency and amplitude, respectively, in the three stages were p = 0.24; p = 0.80; p = 0.44 and p = 0.34; p = 0.78; p = 0.81. For tone-burst stimuli, they were p = 0.43; p = 0.99; p = 0.44 and p = 0.73; p = 0.21; p = 0.26).

Due to the few participants with severe hearing loss (n = 6), it was decided to analyze the P300 latency and amplitude results with the degree of hearing loss. It was observed that the degree of hearing loss did not influence the P300 results – p-values ​​for speech stimuli, for latency and amplitude, respectively, in the three stages were p = 0.97; p = 0.97; p = 0.97 and p = 0.57; p = 0.57; p = 0.12. For tone-burst stimuli, they were p = 0.61; p = 0.62; p = 0.21 and p = 1.00; p = 0.51; p = 0.53. Neither did the configuration of hearing loss – p-values ​​for speech stimuli, for latency and amplitude, respectively, in the three stages were p = 0.50; p = 0.34; p = 0.66 and p = 0.89; p = 0.59; p = 0.69. For tone-burst stimuli, they were p = 0.39; p = 0.86; p = 0.32 and p = 0.88; p = 0.18; p = 0.69.

**Figure 2:**
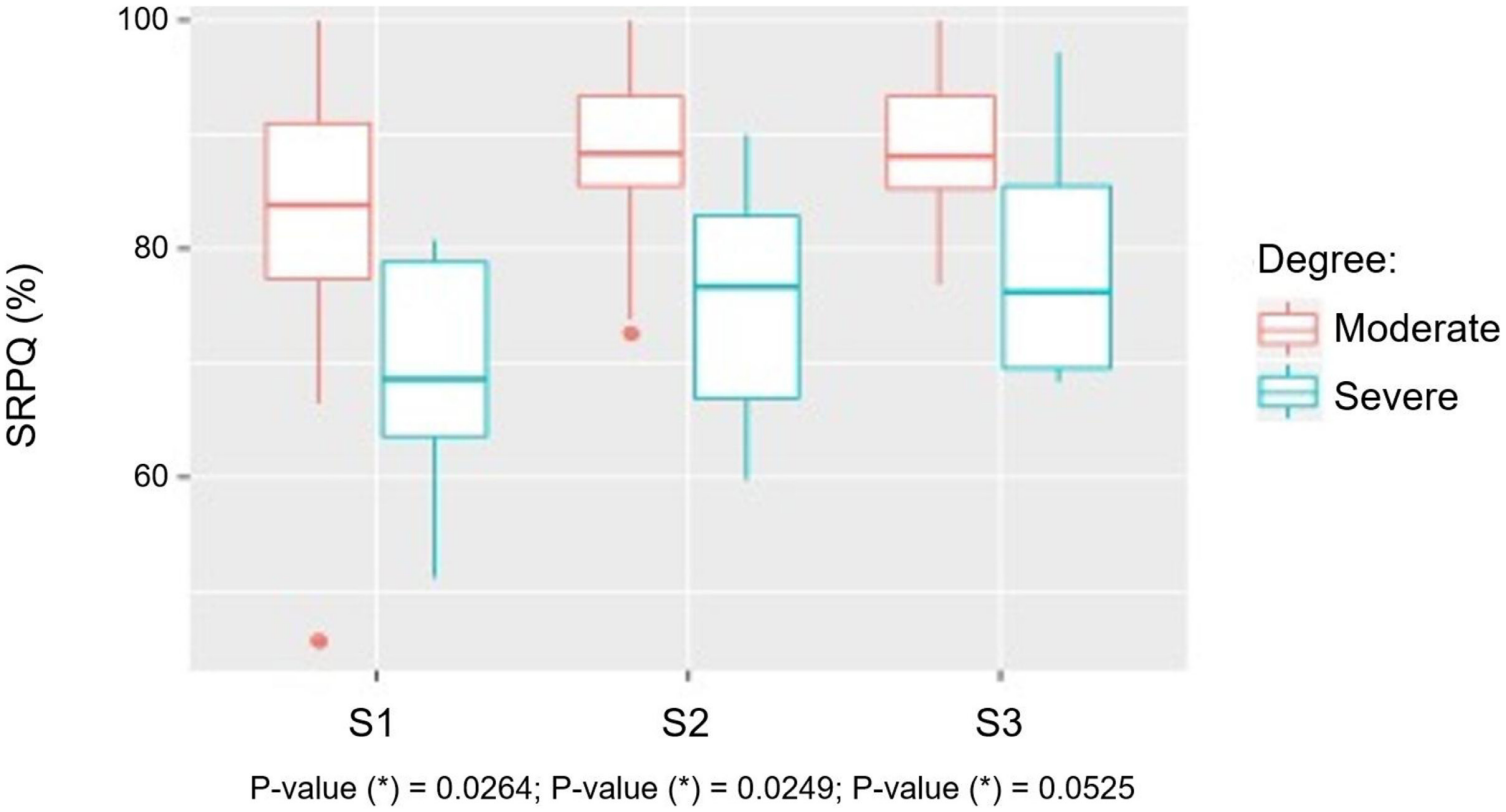
Mean performance results in the sentence recognition percentage index in quiet (SRPQ) in relation to the degree of hearing loss in the three stages of the study

The SRPQ results (Stage 1 = 79%, Stage 2 = 85.4%, and Stage 3 = 86.6%) were statistically different (p = 0.0003) between stages 1 and 2 and 1 and 3, expected due to HA use. The comparison of stages 2 and 3 shows similar mean values, with no difference. No statistically significant differences were observed between the participants' educational level and the sentence recognition test results (Stage 1, p = 0.81; Stage 2, p = 0.97; Stage 3, p = 0.73) or with the length of hearing loss (Stage 1, p = 0.66; Stage 2, p = 0.36; Stage 3, p = 0.79). The comparison between the degree of hearing loss and performance on the sentence recognition test identified a significant difference between individuals with moderate and severe hearing loss. As shown in Figure 2, individuals with moderate hearing loss performed better in stages 1 and 2 (p = 0.0264; p = 0.0249). There was no difference in stage 3 (p = 0.0525), although individuals with moderate hearing loss performed better in the test with competing noise. Hearing loss configuration did not interfere with sentence recognition test results (Stage 1, p = 0.25; Stage 2, p = 0.25; Stage 3, p = 0.24).

**Caption:** S1 = Stage 1, before hearing aid fitting; S2 = Stage 2, six months after hearing aid fitting; S3 = Stage 3, twelve months after hearing aid fitting; SRPQ = sentence recognition percentage index in quiet; % = percent. (*) = Mann-Whitney. Adjusted p-value. Source: Developed by the authors

The HHIE/HHIA was applied to all 24 participants during stages 2 and 3, as it is a questionnaire applied when the individual is using amplification. In total, 16 participants answered the HHIE and eight the HHIA. The overall results in stage 2 show that four individuals perceived no participation restriction, 12 had a mild-to-moderate perception, and eight had a severe or significant perception.

In stage 3, two participants went from severe perception to no perception, another two went from severe perception to mild-to-moderate perception, 10 remained with mild-to-moderate perception, two went from mild-to-moderate perception to no perception, and four remained with no perception of participation restriction.

**Figure 3:**
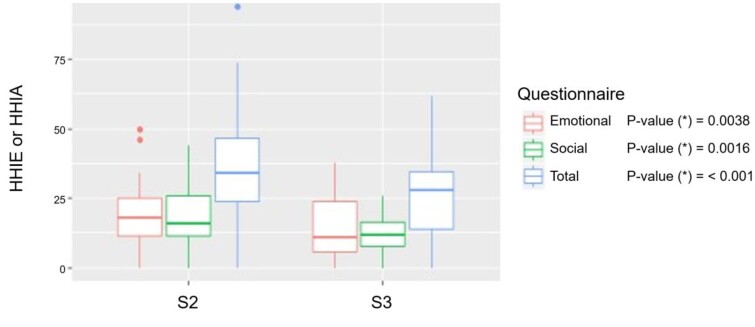
HHIE or HHIA questionnaire at stages 2 and 3

The HHIE/HHIA results are distributed according to the emotional, social, and total domains (Figure 3). We can observe an improvement in all domains in stage 3.

**Caption:** S2 = Stage 2, six months after hearing aid fitting; S3 = Stage 3, twelve months after hearing aid fitting; HHIA = Hearing Handicap Inventory for Adults; HHIE = Hearing Handicap Inventory for the Elderly. (*) = Wilcoxon signed-rank sum. Source: Developed by the authors

## DISCUSSION

The study sample was balanced in terms of sex (45.8% men and 54.2% women). Age ranged from 30 to 78 years, with a mean of 58.9 years (SD+-15.0). There may be age-related variations in the LLAEP P300^(18)^; however, this study controlled this variable, since data collection allowed each individual to be compared with themselves in the three stages. A study^(19)^ showed that P300 latency and amplitude were possibly correlated with brain gray matter volume; its findings support the idea that not only age, but also sex and education level can affect cognitive, neurophysiological, and structural characteristics.

The literature^(20)^ points out that education level also needs to be analyzed in communication situations, since it can significantly predict HA self-efficacy, and that it should be a factor considered during auditory rehabilitation planning. The predominant education level in this research was middle school (n = 12), which is equivalent to 9 years of schooling, a basic education level. No statistical difference was found between the results of the speech perception test and the education level. This raises the hypothesis that research with a balanced sample in terms of educational level, whose participants have attended school for more than 9 years (higher education), may have different results. There was also no statistical difference between the length of hearing deprivation and the results of the sentence recognition test, which suggests that variability in hearing deprivation and education level did not contribute to the results, and especially that the interactions between the peripheral and central auditory systems and the cognitive system are not fully understood.

This study used the LLAEP P300 to look for evidence of functional changes in auditory responses with HAs, since the P300 is a sensitive neural index of cortical processing and reflects the contribution of top-down processes on auditory processing.

The sample consisted of individuals with post-lingual hearing loss, all of whom were able to discriminate between the two types of stimuli used in this research (tone burst and speech). However, during the test application, we noted the need for more orientation time for the individual to clearly perceive and discriminate the rare speech stimulus (/da/). We clarify that the participants did not undergo formal auditory rehabilitation therapy during the study period.

As this study was conducted in three different stages, stability of the response for the electrophysiological assessment was necessary. The literature addresses P300 test-retest in normal-hearing individuals, indicating that the P300 latency is reliable in this condition^(21)^. It also highlights that the stability of the P300 latency result does not reveal a difference between the test-retest when the examinations are performed on the same patients and in a short time^(22)^. Even though the test-retest time in the present study was considered long (6 months and 12 months), and participants had undergone auditory rehabilitation (assuming reorganization of the central auditory pathway), the test and retest results follow the same patterns when compared with normal-hearing individuals.

The P300 is known to allow researchers to observe substrates of neurophysiological processes related to cognition that occur in the cortex. These involve memory and attention, both of which are necessary for central auditory processing^(23)^. This study did not analyze memory because the P300 recording task did not require the patient to mentally count the rare stimuli, but rather to raise their index finger. Thus, we stimulated executive function, which requires planning and executing actions.

It was expected that the average P300 latencies in this study would gradually improve for tone-burst and speech stimuli – i.e., shorter latencies in subsequent phases, due to more experience with acoustic stimulation in HA use in stages 2 and 3. However, this did not occur. After 6 months of HA use, the P300 showed better and worse results among individuals. However, after 12 months, all values ​​were better (lower) compared to stage 1. Average latencies remained within normal levels during all stages, according to the literature^(5)^. Individuals with better auditory recognition are known to have shorter latencies^(24)^, which was also noted in this study – i.e., latencies decreased when speech perception performance results improved.

The average P300 latency results for speech stimuli were always lower than those for tone-burst stimuli in all three stages. Studies^(25)^ suggest that the brains of people with hearing loss process speech stimuli more accurately and efficiently when using HAs. A study with normal-hearing individuals^(26)^ found opposite results when comparing tone burst and speech. It is well reported that speech stimuli present a more complex task when compared to nonverbal stimuli; therefore, the P300 can document slower neural processing for speech stimuli^(27)^. Thus, the most likely hypothesis is that we would find longer P300 latencies for a more complex stimulus, such as speech. However, the opposite occurred in this study, which raises the hypothesis that, since speech was the most difficult stimulus, individuals paid more attention to it during syllable discrimination training and then performed the test with a higher level of attention, which likely required greater cognitive activity and caused them to exhibit faster responses to speech stimuli. Another fact to consider regarding the latency results is that all individuals in this sample had postlingual deafness and, therefore, this auditory pathway was already established at some point. Moreover, all had moderate to severe hearing loss, so most of them still maintained some auditory input in the cortex, even before HA fitting. This hypothesis can be verified with the results of the sentence recognition test and the degree of hearing loss, showing that behavioral and electrophysiological measures are related to the degree of hearing loss, stimulus intensity, and cortical auditory processing.

Electrophysiological assessment, especially the P300, may not be a sensitive test for observing auditory functional plasticity in postlingual individuals 12 months after HA fitting, as we did not gather statistical evidence of it in this study. Since we compared behavioral tests, these showed more significant results than electrophysiology, possibly due to the auditory (detection/discrimination) and cognitive (attention) skills involved. Participants may not have required relevant functional changes to the point of being identified with the P300.

The ANOVA-RM test was used at different stages to check if the type of stimulus (speech and tone burst) had any influence on the results. We found no statistical evidence in these comparisons. The literature seldom reports any influence of the type of stimulus, especially with HA users. As previously mentioned, the brains of people with hearing loss process speech stimuli more accurately and efficiently when equipped with HAs. Thus, the P300 can be useful to clarify the changes in auditory perception that occur due to sound amplification. In a study with normal-hearing individuals^(28)^, researchers found differences in P300 latency between different stimuli (lower latency with tone burst), although such a difference did not occur with amplitude levels.

The amplitude values ​​we found were relatively low during all stages, close to 4 µV. We found that amplitude values ​​improved for the tone burst as the stages progressed and more auditory stimulation time passed. With speech stimuli, the amplitude decreased over time. According to some authors^(27)^, the P300 amplitude is greater for easier tasks, decreasing as the task becomes more difficult.

The latency and amplitude results were not significantly different between the stages. However, during the electrophysiological test, wave morphology seemed to favor the markings of the N1-P2-N2 complex, and particularly the P300 in the rare recording, as the stages progressed.

It is worth noting that the P300 showed latency and amplitude improvement after some time of auditory stimulation with HAs. This highlights the importance of using this tool to monitor the functional neuroplasticity of the central auditory nervous system in HA users. Considering P300’s low sensitivity to detect subtle functional changes in individuals with postlingual hearing loss who use HAs, it is suggested that future research use samples with more participants.

The statistical analysis showed no statistical differences between the P300 and the education level, length of auditory deprivation, degree of hearing loss, or configuration of hearing loss. Therefore, even though six individuals presented with severe hearing loss, there was no influence of hearing loss on the P300 latency, indicating that peripheral hearing loss does not prevent them from performing this test, provided they have the auditory capacity to detect and discriminate the stimulus. The literature mentions^(6,29)^ that the P300 is not affected by hearing loss, as long as the individual can perceive the stimulus. No differences were expected between the degree of hearing loss and the P300 latency and amplitude in this study, since the participants were postlingual and had guaranteed access to auditory stimulation (frequency and intensity), as we used 30 dB HL. It is worth highlighting the care that must be taken in individuals with hearing loss to avoid reaching the auditory discomfort threshold, as this could affect the test results.

The average percentage of SRPQ scores in the sentence recognition test was higher when individuals were assessed with Has (i.e., in stages 2 and 3). The difference found between stages 1 and 2 and stages 1 and 3 was expected, since stages 2 and 3 involved the use of amplification.

Answering the test during the SRPN research in stage 3 became more difficult due to the added noise. Participants reported that the noise made the sentences unintelligible. They could detect the speech but did not recognize complete sentences or parts of them. Even though all individuals had difficulties, those with moderate hearing loss had better results.

The method with competing noise posed a challenge for the participants, since the SRTQ calculated thresholds at which they recognized about 50% of the speech stimuli. Thus, low average scores were expected for this type of situation. The search method used, with intervals of 10 dB and 5 dB, may have limited the responses, as these are relatively wide intervals. We believe it is important to use shorter intervals, such as 4 dB and 2 dB.

The statistical analysis showed no statistical differences between the sentence recognition test and educational level, hearing deprivation time, or hearing loss configuration. However, the degree of hearing loss influenced the results – i.e., individuals with moderate hearing loss performed better in stages 1 and 2 than those with severe hearing loss. There was no difference in stage 3, possibly due to the time of auditory stimulation with HAs.

The questionnaire data suggest that HA use, when properly fitted, tends to improve quality of life, as it reduces the effects of hearing loss on social relationships and communication. Hearing has a significant influence on quality of life, since family and social isolation can worsen cases of depression and social isolation. This study demonstrated a positive impact of HA use on quality of life, as it reduced perceived participation restriction in emotional and social contexts. Most of the sample (18 individuals), despite not changing categories, had better total scores in stage 3. Thus, the HHIE/HHIA showed favorable results as early as 6 months and, especially, 12 months after fitting. This corroborates another study^(9)^, which reports improvement in questionnaire scores after the intervention. Thus, the combination of electrophysiological and behavioral assessments and questionnaire application helped to better understand auditory performance.

## CONCLUSION

The P300 electrophysiological examination in this study did not indicate evidence of auditory neuroplasticity in adults with postlingual sensorineural hearing loss after intervention with HA use. On the other hand, the findings demonstrated improved speech perception and reduced restriction of participation in social and emotional contexts.

This reinforces the importance of using HAs not only in auditory rehabilitation but also in the impact it has on promoting quality of life and social inclusion. Moreover, clinical approaches in rehabilitation monitoring should consider the contribution of systematically applied sentence tests with and without noise and assessment questionnaires.
