# Supplementary material for: Hearing aid fitting: analysis of the impact on the auditory system during the first year of use in moderate and severe hearing loss
Source: Codas. 2026 Jun 15;38(4):e20250077. doi: 10.1590/2317-1782/e20250077en (PMC13322488; doi:10.1590/2317-1782/e20250077en)
Supplement: Translation

# Translation: Adaptação de aparelho de amplificação sonora individual: análise do impacto no sistema auditivo durante o primeiro ano de uso em perdas auditivas moderada e severa

**DOI:** 10.1590/2317-1782/e20250077pt

**Authors:** Victor Goiris Calderaro, Maria Stella Arantes do Amaral, Eduardo Tanaka Massuda, Ana Cláudia Mirândola Barbosa Reis

**Affiliations:** 1 Centro Especializado de Otorrinolaringologia e Fonoaudiologia, Hospital das Clínicas de Ribeirão Preto, Faculdade de Medicina de Ribeirão Preto – FMRP, Universidade de São Paulo – USP - Ribeirão Preto (SP), Brasil.; 2 Departamento de Oftalmologia, Otorrinolaringologia e Cirurgia de Cabeça e Pescoço, Faculdade de Medicina de Ribeirão Preto – FMRP, Universidade de São Paulo – USP - Ribeirão Preto (SP), Brasil.; 3 Departamento de Ciências da Saúde, Faculdade de Medicina de Ribeirão Preto – FMRP, Universidade de São Paulo – USP - Ribeirão Preto (SP), Brasil.

**Keywords:** Perda Auditiva, Auxiliares de Audição, Potencial Evocado P300, Percepção da Fala, Inquéritos e Questionários

## Abstract

**Objetivo:**

Investigar o comportamento do sistema auditivo em indivíduos que fazem uso de aparelhos de amplificação sonora individual, ao longo do primeiro ano de uso.

**Método:**

Participaram do estudo 24 indivíduos, de ambos os sexos, com perda auditiva sensorioneural moderada ou severa. A primeira etapa de coleta ocorreu antes da adaptação dos aparelhos de amplificação sonora individual, mediante a aplicação do exame eletrofisiológico P300 e do teste Listas de Sentenças em Português. A segunda e terceira etapas ocorreram 6 e 12 meses após o início da reabilitação, sendo aplicados os dois testes mencionados e o questionário*Hearing Handicap Inventory for Adults*ou*Hearing Handicap Inventory for The Elderly*.

**Resultados:**

Não houve diferença na latência e amplitude do P300 entre as etapas, tanto quando realizado com tone burst, como para estímulo de fala. Os limiares auditivos pós adaptação permitiram acessibilidade aos sons da fala, diferindo, portanto, a etapa 1 das etapas 2 e 3, sem diferença entre as duas últimas. Houve evidência de melhora na percepção da fala ao longo das três fases, principalmente nos indivíduos com perda auditiva de grau moderado. O questionário de autoavaliação mostrou que o uso de aparelhos de amplificação sonora individual reduziu a restrição de participação percebida em contextos emocionais e sociais.

**Conclusão:**

Embora não haja alterações nas medidas eletrofisiológicas ao longo do primeiro ano de uso, observou-se melhora na percepção da fala (reconhecimento). Além disso, os usuários relatam uma melhora na qualidade de vida, evidenciando os benefícios do uso de aparelhos de amplificação sonora individual.

## INTRODUÇÃO

A privação auditiva impacta a vida de uma pessoa em sua capacidade de perceber estímulos, se relacionar com os outros e transmitir ideias e sentimentos. Além disso, estudos têm mostrado que o tempo de privação auditiva pode estar associado ao declínio cognitivo e à reorganização cortical intermodal, especialmente nos idosos^(1,2)^. Após o diagnóstico de perda auditiva, o uso de dispositivos eletrônicos aplicados à surdez, o que inclui Aparelhos de Amplificação Sonora Individual (AASI), implantes cocleares e próteses auditivas ancoradas ao osso, tem se apresentado como intervenções eficazes e promissoras. O AASI é a opção mais utilizada mundialmente e sua reintrodução para a estimulação auditiva pode levar a mudanças funcionais no sistema responsável pela informação auditiva^(3)^. Atualmente, os testes objetivos têm se tornado populares para avaliar o sistema auditivo e monitorar o processo de reabilitação. Apesar de não existir um único procedimento que possa responder a perguntas sobre a integridade funcional do sistema auditivo central^(4)^, a combinação de testes objetivos e comportamentais pode informar sobre os processos neurais envolvidos na decodificação.

Incluir exames eletrofisiológicos da audição na prática clínica é interessante a medida que os potenciais evocados auditivos permitem avaliar todo o sistema auditivo, desde o órgão sensorial auditivo até as áreas subcorticais e corticais^(5)^.

O Potencial Evocado Auditivo de Longa Latência (PEALL) P300 tem sido considerado uma ferramenta promissora para monitorar intervenções, pois pode ser registrado em indivíduos com perda auditiva, que conseguem acessibilidade e discriminar os estímulos^(6)^. O P300 é um marcador eletrofisiológico relacionado a processos cognitivos, como atenção e discriminação auditiva, sendo analisado pelas medidas de latência e amplitude^(5)^. Em usuários de AASI, sua análise permite investigar o comportamento do sistema auditivo central decorrente da estimulação sonora, complementando a avaliação perceptiva tradicional. O uso do AASI pode promover a plasticidade das vias auditivas centrais aos 6 meses após a adaptação, aumentando o número de neurônios responsivos aos estímulos sonoros^(7)^.

Juntamente aos testes eletrofisiológicos, a avaliação comportamental da percepção da fala com sentenças, nos permite verificar o desempenho auditivo em situações de comunicação. A inteligibilidade da fala de usuários de AASI é favorecida por ambientes silenciosos, com uma baixa relação sinal ruído. Assim, testes de reconhecimento de fala com ruído competitivo vem sendo elaborados para avaliar pessoas com perda auditiva, pois verificam as habilidades auditivas em condições que se assemelham a situações da vida diária^(8)^.

Além dos testes eletrofisiológicos e comportamentais, o uso combinado de questionários que avaliam como os usuários de AASI percebem a restrição de participação em situações cotidianas, pode trazer uma compreensão detalhada dos efeitos da perda auditiva nos aspectos de comunicação e qualidade de vida^(9)^. Assim, utilizar questionários padronizados permite estabelecer as possíveis necessidades e expectativas dos pacientes, quantificar a melhora após a intervenção^(10)^ e a validar decisões na prática audiológica.

Existem poucos estudos na literatura utilizando o PEALL P300 em pacientes com AASI bilateral, recém-adaptados. Por outro lado, há uma tendência de combinar testes comportamentais e objetivos para monitoramento. Ao considerar a possibilidade de entender as dificuldades enfrentadas na adaptação, e promover, quando necessário, a reavaliação e o replanejamento do processo, o objetivo deste estudo foi investigar como o sistema auditivo se comporta ao longo do primeiro ano de uso de AASI bilateral, em adultos com perda auditiva pós-lingual, de grau moderado e severo, considerando aspectos da percepção auditiva comportamental, eletrofisiológica e da autopercepção relatada.

## MÉTODO

Foi realizado um estudo observacional, descritivo, longitudinal, comparativo, com ênfase em pesquisa diagnóstica. Este estudo foi aprovado pelo comitê de ética em pesquisa do Hospital das Clinicas da Faculdade de Medicina de Ribeirão Preto da Universidade de São Paulo, sob número de protocolo HCRP 8774/2017. Os procedimentos de coleta de dados tiveram início somente após a assinatura do Termo de Consentimento Livre e Esclarecido por todos os participantes.

### Participantes

Participaram 24 indivíduos com perda auditiva sensorioneural simétrica, bilateral, sendo 18 de grau moderado e seis de grau severo^(11)^, de ambos os sexos, sendo 11 homens e 13 mulheres, com idades entre 30 e 78 anos, com média de 58,9 anos (DP ±15,0). A escolha dessa faixa etária teve como objetivo representar a diversidade de usuários de AASI atendidos em ambiente clínico. A idade de aquisição da perda auditiva variou de 9 a 65 anos (média de 46,54 anos) e o tempo de privação auditiva variou de 2 a 31 anos (média de 12,37 anos). Quanto à etiologia da perda auditiva, 21 desses indivíduos estavam sob investigação, um foi diagnosticado com perda auditiva induzida por ruído e dois com presbiacusia.

Como critérios de inclusão, considerou-se indivíduos de 18 a 79 anos, de ambos os sexos, com perda auditiva sensorioneural bilateral, simétrica, de moderada a severa, sem histórico de uso de qualquer tipo de dispositivo eletrônico aplicado à surdez. Os critérios de exclusão abrangeram indivíduos diagnosticados com distúrbios neurológicos e/ou psiquiátricos, perdas auditivas com componentes condutivos e/ou mistos, e os que não quiseram concluir o processo de avaliação. Para controlar os critérios de exclusão, utilizou-se o Rastreio Cognitivo de 10 Pontos (10-CS) em indivíduos com mais de 60 anos^(12)^. Embora não tenham sido aplicadas provas específicas de competência linguística, o rastreio cognitivo utilizado incluiu itens de orientação e compreensão verbal, permitindo garantir que os participantes apresentavam condições cognitivas e comunicativas adequadas para a realização das tarefas propostas.

### Etapas e procedimentos

Inicialmente, os participantes foram entrevistados para obtenção dos dados de identificação e demografia, o que inclui idade, nível de escolaridade, nível sociocultural e ocupação, assim como, aplicado o Rastreio Cognitivo de 10 Pontos (10-CS). Também foram consultados os prontuários clínicos para obtenção de informações audiológicas, incluindo a classificação da perda auditiva, tempo de privação auditiva e etiologia da perda. Posteriormente, os dados de pesquisa foram coletados em três etapas.

A primeira etapa ocorreu no dia em que os participantes receberam AASI bilateral, anteriormente à adaptação dos mesmos e consistiu na pesquisa dos limiares auditivos em campo livre sem AASI, aplicação do PEALL P300 em campo livre e aplicação do teste Listas de Sentenças em Português (LSP)^(13)^. A segunda e terceira etapas ocorreram seis e 12 meses (± um mês) após a adaptação de AASI, os quais foram adaptados com a regra prescritiva NAL-NL2. As medidas necessárias foram realizadas para garantir que os participantes recebessem a amplificação adequada. Todos utilizaram por, no mínimo, 8 horas diárias, conforme autorrelato. Não foi utilizado o registro dos AASI (datalog), desta forma, sugere-se, para estudos futuros, que se utilize o registro de tempo (*datalog*) presente no software de programação, para registrar com exatidão o tempo de uso. Nessas etapas realizou-se a pesquisa dos limiares auditivos em campo livre com e sem AASI, aplicação do PEALL P300 em campo livre com AASI, aplicação do teste LSP e aplicação do questionário *Hearing Handicap Inventory for Adult* (HHIA)^(14)^ ou *Hearing Handicap Inventory for The Elderly* (HHIE)^(15)^, de acordo com a idade do indivíduo.

Para a pesquisa de limiar auditivo em campo livre foi utilizado o audiômetro Madsen Astera 2-Otoflex e um alto-falante, dentro da cabine audiométrica. A pesquisa do limiar de campo livre foi conduzida em 500 Hz, 1, 2, 3 e 4 kHz. Durante o procedimento, o participante ficou sentado a 80 cm de distância do alto-falante, posicionado a 0° de azimute, na altura da orelha. Os limiares foram pesquisados com a técnica descendente-ascendente, utilizando estímulo *warble* e o indivíduo foi solicitado a levantar a mão ao ouvir o estímulo.

No exame eletrofisiológico, foi utilizado o PEALL P300, realizado no equipamento Navigator® Pro da Bio-logic® Systems Corp. Potencial Evocado Auditivo de dois canais – AEPSystem, versão 1.3.0 / Natus Medical, EUA, conectado a um computador convencional. Foram utilizados os seguintes parâmetros de registro: ganho de 50.000 µV, filtro 30.0 (alta frequência) e 1.0 (baixa frequência), tempo de análise de 520 ms e polaridade rarefeita. A composição dos eletrodos se fez com o eletrodo ativo posicionado em Cz (jumper, canais 1 e 2, entrada 1), eletrodos de referência nos lóbulos da orelha esquerda (A1) e da direita (A2) (entrada 2) e eletrodo terra em Fpz. Aceitou-se uma impedância individual dos eletrodos ≤ 5kΩ e entre eletrodos ≤ 2KΩ.

O teste foi realizado em campo livre, em ambiente acusticamente controlado, com o participante semi-sentado em uma poltrona reclinável. A caixa de som foi colocada a 1 metro de distância, 0º azimute, na altura do pavilhão auditivo.

Os participantes foram orientados a manter os olhos abertos e fixos em um ponto específico à sua frente para evitar artefatos e ao ouvir os estímulos, levantando o dedo indicador ao identificar apenas o estímulo raro. Os estímulos utilizados foram *tone burst* e estímulo de fala. Para garantir os princípios de percepção acústica dos estímulos apresentados, ou seja, viabilizar o princípio de igualdade à condição dos participantes desta amostra quanto à intensidade do estímulo, foi utilizado 30 dB NS. Desta forma, para o estímulo *tone burst*, apresentado em 1k Hz (estímulo frequente) e 2k Hz (estímulo raro), o nível de sensação utilizado para a apresentação destes estímulos foi de 30 dB acima do limiar psicoacústico encontrado em campo livre para estas frequências. Para o estímulo de fala foram utilizadas as sílabas /ba/ (estímulo frequente) e /da/ (estímulo raro), já presentes sinteticamente no equipamento. O nível de sensação utilizado para a apresentação destes estímulos foi de 30 dB NS, a partir da média tritonal (0.5k, 1k e 2k Hz) do limiar psicoacústico, em campo livre.

Ambos os estímulos utilizados foram apresentados em ordem aleatória e imprevisível, na probabilidade de 20% de estímulos raros (40 estímulos) e 80% de estímulos frequentes (aproximadamente 200 estímulos). Para confiabilidade do registro eletrofisiológico, dois rastreios sucessivos foram aplicados, com objetivo de replicar as ondas. Também foi observada a relação entre identificar o estímulo raro ao levantar o dedo indicador e o aparecimento real do estímulo, com objetivo de descartar a falta do registro do componente P3 pela falta de atenção do participante. O registro com melhor morfologia de onda foi analisado.

Para análise da percepção de fala foi utilizado o teste LSP, que compreende 7 listas para medir as habilidades de reconhecimento de fala. Uma das listas (1A) tem 25 frases e as outras seis (1B, 2B, 3B, 4B, 5B, 6B) têm 10 frases cada. As listas são semelhantes em termos de contexto fonético e estrutura da frase, tornando-as equivalentes. Palavras com significado lexical e semântico, como substantivos, adjetivos e verbos, foram consideradas palavras-chave e pontuadas com dois pontos. Palavras com significado gramatical, como preposições e conjunções, pontuam um ponto. Como as listas têm contagens de palavras diferentes, a pontuação total foi multiplicada por um valor de correção, obtendo pontuação máxima de 100%^(13,16)^.

Primeiramente, o Limiar de Reconhecimento de Sentenças no Silêncio (LRSS) foi obtido, ele indica a intensidade necessária para que o indivíduo reconheça cerca de 50% dos estímulos. O teste foi conduzido em um ambiente de campo livre controlado, onde o indivíduo estava sentado em frente à fonte sonora, na altura da orelha e a 0° azimute. Nesse momento, as sentenças da lista 1A foram utilizadas. Para obter o LRSS, as frases foram apresentadas de acordo com a estratégia ascendente-sequencial^(17)^. Dessa forma, uma vez que o indivíduo respondesse corretamente a uma frase inteira, a seguinte era apresentada em uma intensidade menor. Se ele não respondesse corretamente, a intensidade era aumentada. Inicialmente, utilizou-se intervalos de 10 dB até que o indivíduo errasse, então utilizou-se intervalos de 5 dB até que o LRSS fosse encontrado.

Após a intensidade do LRSS de cada indivíduo ser encontrada, ela foi mantida fixa para obter o Índice de Percentual de Reconhecimento de Sentenças no Silêncio (IPRS), o qual é a soma de todas as palavras repetidas corretamente em cada uma das 10 sentenças, multiplicada pelo valor de correção para obter um número percentual. As pontuações variam de 0 (desempenho mínimo) a 100% (máximo). Utilizou-se as listas 1B (1ª etapa), 3B (2ª etapa) e 5B (3ª etapa).

Na etapa 3, além de LRSS e do IPRS, também se calculou o Índice de Percentual de Reconhecimento de Sentenças no Ruído (IPRSR), utilizando a lista 6B. Nesse teste, um ruído foi apresentado simultaneamente às sentenças. A relação sinal ruído foi mantida em -5 dB, com a fala sendo apresentada a 0° e o ruído a 180°. A intensidade da fala utilizada foi a mesma do LRSS.

O questionário HHIA/HHIE foi aplicado durante as etapas 2 e 3 para caracterizar a restrição de participação, uma vez que, este questionário é utilizado apenas quando o indivíduo está em uso dos dispositivos eletrônicos aplicados à surdez. Possui 25 questões, divididas em duas escalas, sendo uma social/situacional e a outra emocional. O questionário foi utilizado como roteiro dirigido e administrado como uma entrevista direta, o participante foi instruído a escolher uma resposta para cada pergunta, sendo: sim (4 pontos), às vezes (2 pontos) ou não (0 pontos). A pontuação total é calculada somando os pontos. Quanto maior a pontuação, maior a restrição auto percebida, sendo que menos de 16 pontos significa a não percepção de restrição de participação, de 18 a 42 significa uma restrição leve e acima de 42 pontos significa uma restrição de participação severa ou significativa.

### Análise dos dados

Os resultados qualitativos foram apresentados como frequência e proporção. Os resultados quantitativos foram apresentados como média e desvio padrão (média ± DP) quando métodos paramétricos foram utilizados, e mediana (intervalo interquartílico, IQR) quando avaliados com métodos não paramétricos. Utilizamos análise de variância com medidas repetidas (ANOVA-RM) para comparar diferenças na latência (ms) e amplitude (µV) entre as fases. Medidas dependentes foram comparadas com os testes de Wilcoxon e t de Student. Para medidas independentes, utilizou-se os testes de Mann-Whitney e Kruskal-Wallis. As correlações entre as variáveis foram avaliadas com o teste de Tukey-HSD ou tiveram o valor de p corrigido com o método da Taxa de Descoberta Falsa (FDR). A significância de p < 0,05 foi utilizada.

## RESULTADOS

Na pesquisa do limiar auditivo em campo livre foram encontrados limiares auditivos médios para as três etapas, sendo: Etapa 1 = 55,1 dB NPS; Etapa 2 = 36,1 dB NPS e Etapa 3 = 36,9 dB NPS. Como esperado, devido ao benefício do uso de AASI, há uma diferença estatística (p < 0,001) entre as etapas 1 e 2 e as etapas 1 e 3 ao usar AASI. Na comparação entre as fases 2 e 3, os resultados são semelhantes, sem diferença estatística.

Na Tabela 1 estão demonstradas as médias e desvio padrão das medidas de latência e amplitude do P300 para todos os 24 participantes, em todas as etapas e para ambos os estímulos utilizados. É possível observar que as latências para o estímulo de fala foram menores nas três etapas, em comparação ao estímulo *tone burst,* mas as amplitudes foram semelhantes. Independente do tipo de estímulo utilizado, as médias de latência estão dentro do esperado como padrão de normalidade do P300.

**Tabela 1:** Médias das medidas de latência (ms) e amplitude (µV) do potencial P300 nas três etapas do estudo, tanto para estímulos *tone-burst* quanto para estímulos de fala

**Estímulo**	**Etapas**	**Latência (ms)**		**Amplitude (µV)**	
		**Média**	**DP**	**Média**	**DP**
*Tone-burst*	1	348,85	51,5	3,38	2,6
	2	334,84	37,1	4,12	2,8
	3	337,01	40,6	4,35	2,2
Fala	1	326,08	27,6	4,25	2
	2	330,72	27,1	4,26	1,8
	3	318,62	29,6	3,70	2,1

**Legenda:** DP = Desvio Padrão; ms = milissegundos; µV = microvolts. Fonte: elaborada pelos autores

Na Figura 1 pode-se observar as médias das medidas de latências do P300 para todas as três etapas, considerando os estímulos *tone burst* e estímulos de fala. A análise estatística indicou, ao comparar a etapa sem AASI com as etapas em que o indivíduo estava em uso de AASI (E1xE2 e E1xE3), que não houve diferença estatística (p = 0,35 e p = 0,20, respectivamente). Da mesma forma, os valores médios das medidas de amplitude do P300, usando estímulos *tone burst* e estímulos de fala, não mostraram nenhuma diferença (p = 0,057 e p = 0,12, respectivamente), conforme observado na Figura 1.

**Figura 1:** Valores médios das medidas de latência (ms) e amplitude (µV) do P300 para os estímulos *tone-burst* (a) e de fala (b) nas três etapas do estudo

**Legenda:** E1 = Etapa 1, antes da adaptação do AASI; E2 = Etapa 2, seis meses após a adaptação do AASI; E3 = Etapa 3, doze meses após a adaptação do AASI; ms = milissegundo; µV = microvolt. (*) = Análise de Variância para medidas repetidas (ANOVA-RM). Fonte: elaborada pelos autores

Optou-se por realizar uma análise entre o nível de escolaridade e os resultados de latência e amplitude do P300, a qual não foi observada diferença estatística (valores de p para o estímulo de fala, para latência e amplitude, respectivamente, nas três etapas: p = 0,49; p = 0,86; p = 0,8 e p = 0,49; p = 0,86; p = 0,81. Para o estímulo *Tone burst:* p = 0,91; p = 0,04; p = 0,06 e p = 0,79; p = 0,10; p = 0,54). Além disso, não houve evidência estatística entre latência e amplitude do P300 com o tempo de privação auditiva dos participantes, para as três etapas (valores de p para o estímulo de fala, para latência e amplitude, respectivamente, nas três etapas: p = 0,24; p = 0,80; p = 0,44 e p = 0,34; p = 0,78; p = 0,81. Para o estímulo *Tone burst:* p = 0,43; p = 0,99; p = 0,44 e p = 0,73; p = 0,21; p = 0,26).

Devido ao baixo número de indivíduos com perda auditiva de grau severo (n=6), optou-se por analisar os resultados de latência e amplitude do P300 com o grau de perda auditiva e observou-se que não houve influência do grau de perda nos resultados do P300 (valores de p para o estímulo de fala, para latência e amplitude, respectivamente, nas três etapas: p = 0,97; p = 0,97; p = 0,97 e p = 0,57; p = 0,57; p = 0,12. Para o estímulo *Tone burst:* p = 0,61; p = 0,62; p = 0,21 e p = 1,00; p = 0,51; p = 0,53), assim como, com a configuração da perda auditiva (valores de p para o estímulo de fala, para latência e amplitude, respectivamente, nas três etapas: p = 0,50; p = 0,34; p = 0,66 e p = 0,89; p = 0,59; p = 0,69. Para o estímulo *Tone burst:* p = 0,39; p = 0,86; p = 0,32 e p = 0,88; p = 0,18; p = 0,69).

Os resultados do IPRSS nas três etapas (Etapa 1 = 79%, Etapa 2 = 85,4% e Etapa 3 = 86,6%) mostram uma diferença estatística (p = 0,0003) entre as etapas 1 e 2 e 1 e 3, esperado devido ao uso de AASI. A comparação das etapas 2 e 3 mostra valores médios semelhantes, sem diferença. Também não foram observadas diferenças estatísticas entre o nível de escolaridade dos participantes e os resultados do teste de reconhecimento de sentenças (Etapa 1, p = 0,81; Etapa 2, p = 0,97; Etapa 3, p = 0,73), nem tão pouco com o tempo de privação auditiva (Etapa 1, p = 0,66; Etapa 2, p = 0,36; Etapa 3, p = 0,79). A comparação entre o grau de perda auditiva e o desempenho no teste de reconhecimento de sentenças identificou uma diferença significativa entre indivíduos com perda auditiva moderada e severa. Conforme mostrado na Figura 2, indivíduos com perda auditiva moderada tiveram um melhor desempenho nas etapas 1 e 2 (p = 0,0264 / p = 0,0249). Não houve diferença na etapa 3 (p = 0,0525), embora, ao realizar o teste com ruído competitivo, os indivíduos com perda auditiva de grau moderado alcançaram um melhor desempenho. A configuração da perda auditiva não interferiu nos resultados do teste de reconhecimento de sentenças (Etapa 1, p = 0,25; Etapa 2, p = 0,25; Etapa 3, p = 0,24).

**Figura 2:** Resultados médios do desempenho do Índice Percentual de Reconhecimento de Sentenças no Silêncio (IPRSS) em relação ao grau da perda auditiva, nas três etapas do estudo

**Legenda:** E1 = Etapa 1, antes da adaptação do AASI; E2 = Etapa 2, seis meses após a adaptação do AASI; E3 = Etapa 3, doze meses após a adaptação do AASI; IPRSS = Índice Percentual de Reconhecimento de Sentenças no Silêncio; % = por cento. (*) = Mann-Whitney. Valor de p ajustado. Fonte: elaborada pelos autores

O questionário HHIE/HHIA foi aplicado em todos os 24 participantes, durante a segunda e terceira etapa, visto que é um questionário aplicado quando o indivíduo está em uso de amplificação. Ao todo, 16 participantes responderam o HHIE e oito o HHIA. Os resultados totais na etapa 2 mostram que quatro indivíduos não perceberam nenhuma restrição de participação, 12 tiveram uma percepção leve a moderada e oito tiveram uma percepção severa ou significativa.

Na etapa 3, dois participantes passaram de percepção severa para nenhuma percepção, outros dois passaram de percepção severa para percepção leve a moderada, 10 permaneceram com percepção leve a moderada, dois passaram de percepção leve a moderada para nenhuma percepção e quatro permaneceram sem percepção de restrição de participação.

Os resultados do HHIE/HHIA são distribuídos de acordo com os domínios emocionais, sociais e totais (Figura 3). Podemos observar uma melhora em todos os domínios na etapa 3.

**Figura 3:** Questionário HHIE ou HHIA nas etapas 2 e 3

**Legenda:** E2 = Etapa 2, 6 meses após adaptação da prótese auditiva; E3 = Etapa 3, 12 meses após a adaptação do AASI; HHIA = *Hearing Handicap Inventory for Adults*; HHIE = *Hearing Handicap Inventory For The Elderly*. (*) = Soma de postos sinalizados de Wilcoxon. Fonte: elaborada pelos autores

## DISCUSSÃO

A amostra do estudo foi equilibrada em termos de sexo (45,8% homens e 54,2% mulheres). A idade variou de 30 a 78 anos, com média de 58,9 anos (DP+-15,0). Embora possam haver variações relacionadas à idade no PEALL P300^(18)^, neste estudo foi uma variável controlada, visto que a coleta de dados permitiu que cada indivíduo fosse comparado consigo mesmo nas três etapas. Um estudo^(19)^ mostrou uma possível correlação da latência e amplitude do P300 com o volume de massa cinzenta no cérebro e suas descobertas apoiam a ideia de que não apenas a idade, mas também o sexo e o nível de escolaridade podem afetar características cognitivas, neurofisiológicas e estruturais.

De acordo com a literatura^(20)^, o nível de escolaridade também precisa ser analisado em situações de comunicação, uma vez que o nível educacional pode prever significativamente a autoeficácia do AASI e aponta que deve ser um fator considerado durante o planejamento da reabilitação auditiva. Nesta pesquisa, o nível de escolaridade predominante foi o ensino fundamental (n=12), o que equivale a nove anos de escola, um nível de educação básica. Nenhuma diferença estatística foi encontrada entre os resultados do teste de percepção da fala e o nível educacional. Isso levanta a hipótese de que pesquisas com uma amostra equilibrada em termos de nível educacional, cujos participantes tenham frequentado a escola por mais de 9 anos (ensino superior), podem ter resultados diferentes. Também não houve diferença estatística entre o tempo de privação auditiva e os resultados do teste de reconhecimento de sentenças, o que sugere que a variabilidade, tanto na privação auditiva, quanto no nível educacional, não contribuiu para os resultados e, especialmente, que as interações entre os sistemas auditivos periférico, central e o sistema cognitivo não são totalmente compreendidas.

Este estudo utilizou o PEALL P300 para buscar evidências de mudanças funcionais nas respostas auditivas com AASI, uma vez que o P300 é um índice neural sensível do processamento cortical e reflete a contribuição de processos *top-down* sobre o processamento auditivo.

A amostra foi composta por indivíduos com perda auditiva pós-lingual, todos os quais capazes de discriminar os dois tipos de estímulos utilizados nesta pesquisa (*tone burst* e fala). No entanto, durante a aplicação do teste, notamos a necessidade de mais tempo de orientação para o indivíduo perceber e discriminar claramente o estímulo de fala raro (/da/). Esclarecemos que os participantes não realizaram terapia formal de reabilitação auditiva durante o período do estudo.

Como este estudo foi conduzido em três etapas diferentes, a estabilidade da resposta para a avaliação eletrofisiológica foi necessária. Há menção na literatura quanto ao teste-reteste de P300 em indivíduos com audição normal, indicando que a latência do P300 é confiável nessa condição^(21)^. Ressalta-se também que a estabilidade do resultado da latência do P300 não revela diferença entre o teste-reteste quando os exames são realizados nos mesmos pacientes e em um curto período de tempo^(22)^. Mesmo que o tempo de teste-reteste no presente estudo tenha sido considerado longo (6 meses e 12 meses) e com indivíduos que passaram por reabilitação auditiva, pressupondo a reorganização da via auditiva central, os resultados do teste e reteste seguem os mesmos padrões quando comparados com indivíduos com audição normal.

O P300 é conhecido por permitir ao pesquisador observar substratos de processos neurofisiológicos relacionados à cognição que ocorrem no córtex. Estes envolvem memória e atenção, ambos necessários para o processamento auditivo central^(23)^. A memória não foi analisada neste estudo porque a tarefa de gravação do P300 não exigiu que o paciente contasse mentalmente os estímulos raros, mas sim que levantasse o dedo indicador. Portanto, estimulamos a função executiva, que requer planejamento e execução de ações.

Esperava-se neste estudo que as latências médias do P300 melhorassem gradualmente para estímulos *tone burst* e de fala, ou seja, latências mais curtas nas fases subsequentes, devido a mais experiência com estimulação acústica em uso de AASI nas etapas 2 e 3. No entanto, isso não ocorreu. Após 6 meses de uso de AASI, o P300 apresentou resultados melhores e piores entre os indivíduos. No entanto, após 12 meses, todos os valores foram melhores (menores) em relação à etapa 1. As latências médias permaneceram dentro dos níveis normais durante todas as etapas, de acordo com a literatura^(5)^. Indivíduos com melhor reconhecimento auditivo são conhecidos por terem latências mais curtas^(24)^, o que foi igualmente notado neste estudo, ou seja, as latências diminuíram quando os resultados do desempenho da percepção da fala melhoraram.

Os resultados médios da latência do P300 para o estímulo de fala foram sempre menores nas três etapas em relação ao estímulo *tone burst*. Estudos^(25)^ sugerem que o cérebro de pessoas com perda auditiva processa estímulos de fala com maior precisão e eficiência ao usar AASI. Um estudo contendo indivíduos com audição normal^(26)^ encontrou resultados opostos quando *tone burst* e fala foram comparados. É bem relatado que estímulos de fala apresentam uma tarefa mais complexa quando comparados a estímulos não verbais e que assim, o P300 pode documentar um processamento neural mais lento para estímulos de fala^(27)^. Assim, a hipótese mais provável é que encontremos latências do P300 mais longas para um estímulo mais complexo, como a fala. No presente estudo, o oposto aconteceu, o que levanta a hipótese de que, como a fala era o estímulo mais difícil, os indivíduos prestaram mais atenção a ela durante o treinamento de discriminação de sílabas e, em seguida, realizaram o teste com um nível de atenção mais elevado, o que provavelmente exigiu uma maior atividade cognitiva e os fez apresentar respostas mais rápidas ao estímulo de fala. Outro fato a se considerar em relação aos resultados de latência é que todos os indivíduos nesta amostra tinham surdez pós-lingual e, portanto, essa via auditiva já foi estabelecida em algum momento. Além disso, todos tinham perda auditiva de grau moderado a severo, então a maioria deles ainda mantinha alguma entrada auditiva no córtex, mesmo antes da adaptação dos AASI. Essa hipótese pode ser verificada com os resultados do teste de reconhecimento de sentenças e o grau de perda auditiva, mostrando que as medidas comportamentais e eletrofisiológicas estão relacionadas ao grau de perda auditiva, intensidade do estímulo e processamento auditivo cortical.

A avaliação eletrofisiológica, especialmente o P300, pode não ser um teste sensível para observar a plasticidade funcional auditiva em indivíduos pós-linguais 12 meses após a adaptação do AASI, pois não reunimos evidências estatísticas disso neste estudo. Como comparamos os testes comportamentais, estes mostraram resultados mais significativos do que a eletrofisiologia, possivelmente devido às habilidades auditivas (detecção/discriminação) e cognitivas (atenção) envolvidas. Os participantes podem não ter necessitado de mudanças funcionais relevantes a ponto de serem identificadas com o P300.

O teste ANOVA-RM foi usado nas diferentes etapas para verificar se o tipo de estímulo (fala e *tone burst*) teve alguma influência nos resultados. Não encontramos nenhuma evidência estatística nessas comparações. A influência do tipo de estímulo raramente é mencionada na literatura, especialmente com usuários de AASI. Como anteriormente mencionado, o cérebro de pessoas com deficiência auditiva processa estímulos de fala com maior precisão e eficiência quando equipado com AASI. Assim, o P300 pode ser útil para esclarecer as mudanças na percepção auditiva que acontecem pela amplificação sonora. Em um estudo com indivíduos com audição normal^(28)^, os pesquisadores encontraram diferenças na latência do P300 entre diferentes estímulos, em que uma latência menor foi encontrada com *tone burst*, embora tal diferença não tenha ocorrido com os níveis de amplitude.

Em relação à amplitude, os valores que encontramos foram relativamente baixos durante todas as etapas, próximos de 4 µV. Verificamos que, para *tone burst*, conforme as etapas avançavam e mais tempo de estimulação auditiva passava, os valores de amplitude melhoravam. Com estímulo de fala, a amplitude diminuiu com o tempo. De acordo com alguns autores^(27)^, a amplitude do P300 é maior para tarefas mais fáceis, diminuindo conforme a tarefa se torna mais difícil.

Os resultados de latência e amplitude não mostraram diferença significativa entre as etapas. No entanto, durante o teste eletrofisiológico, a morfologia da onda pareceu favorecer as marcações do complexo N1-P2-N2, e particularmente do P300 na gravação rara, conforme as fases progrediam.

Vale a pena notar que o P300 mostrou melhora na latência e amplitude após algum tempo de estimulação auditiva com AASI. Isso destaca a importância de usar essa ferramenta para monitorar a neuroplasticidade funcional do sistema nervoso auditivo central em pacientes usuários de AASI. Considerando-se a baixa sensibilidade encontrada no P300 para detectar mudanças funcionais sutis em indivíduos com perda auditiva pós-lingual e usuários de AASI, sugere-se para futuras pesquisas n amostral com maior número de participantes.

A análise estatística não mostrou diferenças estatísticas entre o P300 e as seguintes variáveis: nível de escolaridade, tempo de privação auditiva, grau de perda auditiva e configuração da perda auditiva. Portanto, mesmo que seis indivíduos apresentassem perda auditiva severa, não houve influência da perda auditiva na latência do P300, o que indica que a perda auditiva periférica não os impede de realizar este exame, desde que tenham capacidade auditiva para detectar e discriminar o estímulo. A literatura menciona^(6,29)^ que o P300 não é afetado pela perda auditiva, desde que o indivíduo consiga perceber o estímulo. Não eram esperadas diferenças entre o grau de perda auditiva e a latência e amplitude do P300 neste estudo, uma vez que os participantes eram pós-linguais e também tinham acesso garantido à estimulação auditiva (frequência e intensidade), por termos utilizado 30 dBNS. Vale ressaltar o cuidado que deve ter, em indivíduos com perda auditiva, para não atingir o limiar de desconforto auditivo, pois isso poderia afetar os resultados do teste.

No teste de reconhecimento de sentenças, a porcentagem média de escore do IPRSS foi maior quando os indivíduos foram avaliados com AASI, ou seja, nas etapas 2 e 3. A diferença encontrada entre as etapas 1 e 2 e as etapas 1 e 3 já era esperada, uma vez que as etapas 2 e 3 contavam com o uso de amplificação.

Durante a pesquisa IPRSR na etapa 3, responder ao teste se tornou mais difícil devido ao ruído adicionado. Os participantes relataram que o ruído tornou as frases ininteligíveis. Eles conseguiam detectar a fala, mas não reconheciam frases completas ou partes delas. Mesmo que todos os indivíduos tivessem dificuldades, aqueles com perda auditiva moderada tiveram melhores resultados.

O método com ruído competitivo representou um desafio para os participantes, já que o LRSS calculava limiares nos quais eles reconheciam cerca de 50% dos estímulos de fala. Assim, eram esperadas pontuações médias baixas para este tipo de situação. O método de busca utilizado, com intervalos de 10 dB e 5 dB, pode ter limitado as respostas, já que estes são intervalos relativamente amplos. Acreditamos ser importante usar intervalos mais curtos, como 4 dB e 2 dB.

A análise estatística não mostrou diferenças estatísticas entre o teste de reconhecimento de sentenças e as seguintes variáveis: nível de escolaridade, tempo de privação auditiva e configuração da perda auditiva. No entanto, o grau de perda auditiva influenciou os resultados, ou seja, indivíduos com perda auditiva moderada tiveram um melhor desempenho nas etapas 1 e 2 do que aqueles com perda auditiva severa. Não houve diferença na etapa 3, possivelmente devido ao tempo de estimulação auditiva com AASI.

Os dados do questionário sugerem que o uso de AASI, quando devidamente adaptado, tende a melhorar a qualidade de vida, pois diminui os efeitos da perda auditiva nos relacionamentos sociais e na comunicação. A audição tem uma influência significativa na qualidade de vida, uma vez que o afastamento familiar e social pode agravar casos de depressão e isolamento social. Este estudo demonstrou um impacto positivo do uso de AASI na qualidade de vida, pois reduziu a restrição de participação percebida em contextos emocionais e sociais. A maioria da amostra (18 indivíduos), apesar de não ter mudado a categoria, apresentou melhores pontuações totais na etapa 3. Dessa forma, o questionário HHIE/HHIA mostrou resultados favoráveis já aos 6 meses e, especialmente, 12 meses após a adaptação. Isto corrobora outro estudo ^(9)^, que relata melhora nas pontuações do questionário após a intervenção. Assim, a combinação do exame eletrofisiológico, avaliação comportamental e aplicação de questionário ajudou a entender melhor o desempenho auditivo.

## CONCLUSÃO

Neste estudo, o exame eletrofisiológico P300 não indicou evidências de neuroplasticidade auditiva em adultos com perda auditiva sensorioneural pós-lingual, após intervenção com uso de AASI. Por outro lado, os achados demonstraram melhora na percepção da fala e redução na restrição de participação em contextos sociais e emocionais.

Reforça-se a importância do uso do AASI não apenas na reabilitação auditiva, mas também no impacto que causa na promoção de qualidade de vida e inclusão social, destacando a necessidade de abordagens clínicas de monitoramento da reabilitação que considerem a contribuição da aplicação sistemática de testes de sentenças sem e com ruído e de questionários de avaliação.
